# Intervenção Paliativa Endovascular no Lactente com Tetralogia de Fallot: Uma Série de Casos

**DOI:** 10.36660/abc.20200148

**Published:** 2021-07-07

**Authors:** Kerli Dreier Kupas, Isabela Oldoni, Juliano Mendes Souza

**Affiliations:** 1 Faculdades Pequeno Príncipe CuritibaPR Brasil Faculdades Pequeno Príncipe, Curitiba, PR - Brasil; 2 Universidade federal do Paraná CuritibaPR Brasil Universidade federal do Paraná (UFPR), Curitiba, PR – Brasil

**Keywords:** Cardiopatias Congênitas, Tetralogia de Fallot, Procedimentos Cirúrgicos Operatórios, Lactente, Procedimento de Blalock Taussig

## Abstract

**Fundamento:**

Tendo em vista os casos de lactentes sintomáticos com Tetralogia de Fallot (TF), baixo peso ao nascimento e anatomia complexa, o implante de *stent* na via de saída do ventrículo direito (VSVD) tem sido indicado alternativamente à cirurgia de Blalock-Taussig (BT).

**Objetivo:**

Avaliar o implante endovascular de *stent* na VSVD como abordagem primária no lactente com TF e não candidato à cirurgia de BT, bem como relatar seus resultados a médio prazo e até a retirada do *stent* na cirurgia corretiva.

**Métodos:**

Entre outubro de 2015 e abril de 2018, uma série de seis lactentes portadores de TF receberam *stents* para desobstrução da VSVD. Os parâmetros hemodinâmicos foram comparados em períodos pré e pós-implante.

**Resultados:**

As medianas de idade e peso no momento do procedimento foram de 146,5 dias e 4,9 kg, respectivamente. O gradiente sistólico máximo diminuiu de 63,5 mmHg para 50,5 mmHg, enquanto o diâmetro dos ramos pulmonares direito e esquerdo aumentou de 3,5 mm para 4,9 mm e 4,3 mm, respectivamente. O índice de Nakata aumentou de 96,5 mm para 108,3 mm; assim como o peso, de 4,9 kg para 5,5 kg. A saturação de oxigênio aumentou de 83,5% para 93%. Houve um caso de migração do *stent* e dois óbitos, um deles devido à embolização do *stent* e o outro não teve relação com o procedimento.

**Conclusões:**

O implante de *stent* na VSVD como procedimento paliativo na TF se mostra uma alternativa promissora para o tratamento de lactentes com má anatomia e baixo peso ao nascimento.

## Introdução

A tetralogia de Fallot (TF) é a cardiopatia congênita mais comum dentre as malformações cardíacas cianóticas, acomete três em cada 10.000 nascidos vivos no mundo, com incidência maior em pacientes do sexo masculino.^1 , 2^ Ela se caracteriza por apresentar quatro anomalias básicas: comunicação interventricular, obstrução da via de saída do ventrículo direito, hipertrofia ventricular direita e dextroposição da aorta.^1 , 2^ A sintomatologia varia conforme o grau da estenose na VSVD e o tamanho da comunicação interventricular, manifestando tipicamente a coloração azul-arroxeada, cansaço às mamadas e crises de hipóxia aliviadas pelo uso da posição de cócoras.^3 , 4^ Isso posto, o diagnóstico pode ser feito com o exame de ultrassonografia morfológica durante o pré-natal e deve ser confirmada pela ecocardiografia fetal ou transtorácica no pós-natal. Em casos inconclusivos ou para avaliação pré-operatória, podem ser solicitados o cateterismo, a ressonância magnética ou a tomografia computadorizada.^3 , 5^

Dentre as possibilidades terapêuticas, está disponível a cirurgia corretiva, que pode ser antecedida por procedimentos paliativos, como é o caso da cirurgia de Blalock-Taussig (BT) e o implante de *stent* (IS) na via de saída do ventrículo direito (VSVD).

O BT, empregado desde 1945 como tratamento inicial de escolha, consiste em estabelecer um *shunt* sistêmico-pulmonar, de maneira invasiva, entre as artérias subclávia e pulmonar. Em neonatos e lactentes com menos de três meses, está associada a inúmeros casos de oclusão de *shunt* , mortalidade e falha na preservação anular da valva pulmonar.^3^ Alternativamente a estes lactentes com baixo peso ao nascimento, portadores de alterações anatômicas complexas e com sintomas de moderados a exacerbados, indica-se o IS na VSVD,^6^ que se destaca por ser uma técnica menos invasiva, de baixa taxa de morbimortalidade e restabelece o desenvolvimento neuronal adequado e qualidade de vida dos pacientes até a cirurgia corretiva definitiva.^6 - 8^

Nesse contexto, o objetivo do estudo foi avaliar o implante endovascular de *stent* na VSVD como abordagem primária no lactente com TF e não candidato à cirurgia de BT e identificar modificações ecocardiográficas nos períodos pré e pós-IS na VSVD até a retirada do *stent* na cirurgia corretiva.

## Métodos

Este estudo foi submetido e aprovado pelo Comitê de Ética com o número CAAE 17443119.3.0000.5580. Nesse sentido, respeitou-se as diretrizes e critérios estabelecidos na Resolução n^o^ 466, de 12 de dezembro de 2012, do Conselho Nacional de Saúde (CNS), preceitos éticos estabelecidos no que se refere a zelar pela legitimidade, privacidade e sigilo das informações. Trata-se de um estudo observacional, descritivo e longitudinal que inclui a busca de dados retrospectivos com abordagem quantitativa em um hospital infantil de grande porte de Curitiba (PR).

Os critérios de inclusão foram: ser portador de TF e ter sido submetido a um procedimento terapêutico paliativo endovascular com IS na VSVD. Por sua vez, os critérios de exclusão foram os seguintes: ter sido submetido ao IS cardíaco por outras cardiopatias congênitas que não a TF, apresentar IS em outra região anatômica cardíaca e possuir outras malformações cardíacas.

O presente estudo tratou de uma série dos seis lactentes consecutivos com TF que realizaram IS na VSVD entre outubro de 2015 e abril de 2018. A análise dos dados, disponíveis nos prontuários, foi feita para as seguintes variáveis:

- Dados sociodemográficos: sexo, idade (em dias).
- Dados antropométricos: peso (em kg).
- Dados clínicos: saturação de oxigênio capilar em porcentagem.
- Dados ecocardiográficos: gradientes sistólicos máximos e médios na VSVD em mmHg; tipo e gravidade da estenose na VSVD; tamanhos dos ramos pulmonares direito e esquerdo, do tronco pulmonar e anel valvar pulmonar em mm; índice de Nakata da artéria pulmonar em mm.
- Dados da prótese endovascular: marca e tamanho (diâmetro em mm x comprimento em mm).
- Dados cirúrgicos: descrição da técnica de implante de *stent* .

Tais variáveis de desfecho foram identificadas em três períodos: pré-implante e pós-operatório imediato do IS na VSVD, bem como no pós-operatório tardio da retirada do *stent* da cirurgia corretiva subsequente.

### Análise estatística

Na análise estatística, o banco de dados foi estruturado em uma planilha do Excel (Microsoft) e foram analisados de forma descritiva. Por conseguinte, os resultados foram expressos por medianas, valores mínimos, valores máximos e percentuais.

## Resultados

Foram incluídos seis lactentes, dos quais três eram do sexo masculino e três do sexo feminino, com a mediana de idade de 146,5 dias no procedimento de implante de *stent* na VSVD, que variava entre 68 e 261 dias. Em contrapartida, no procedimento de retirada da endoprótese, a mediana de idade foi de 367 dias, conforme demonstrado na Tabela 1 .

**Tabela 1:** – Idade dos pacientes e mediana no IS na VSVD, na retirada do *stent* na cirurgia corretiva. Idade dos pacientes no intervalo entre IS na VSVD e retirada do *stent* na cirurgia corretiva

Paciente	Idade no IS na VSVD (dias)	Idade na retirada do *stent* na cirurgia corretiva (dias)	Intervalo entre IS na VSVD e retirada do *stent* na cirurgia corretiva (dias)
A	261	1211	950
B	68	1078	1010
C	151	367	216
D	74	184	110
E	142	200	58
F	170	–	–
Mediana	146,5	367	216

*VSVD: via de saída do ventrículo direito; IS: implante de stent.*

As marcas dos *stents* e seus respectivos tamanhos utilizados na VSVD estão demonstrados na Tabela 2 . A marca predominante foi a Dynamic-Biotronik. Dois pacientes não apresentaram o registro do fabricante da prótese.

**Tabela 2:** – Marca e tamanho da prótese endovascular ( *stent* )

Paciente	Marca do stent	Tamanho do stent (diâmetro (mm) x comprimento (mm))
A	Dynamic-Biotronik	8 x 12
B	Dynamic-Biotronik	8 x 15
C	Woven-NIH	8 x 15
D	Dynamic-Biotronik	7 x 15
E	–	–
F	–	–

A técnica do implante de *stent* empregada nos lactentes está descrita a seguir:

- Paciente sob anestesia geral;
- Punção de veia e artéria femoral direita com colocação de introdutores números 6F e 5F, respectivamente;
- Utilização de guia hidrofílica 0,035” e cateter Judkins (JR), realizado manometria D/E;
- Coletas de gasometrias seriadas para oximetrias e realização de cálculo de fluxo e resistência;
- Realização de cineangiografias com cateter tipo *pigtail* ;
- Troca da guia 0,035” pela guia 0,014”;
- Posicionamento do *stent* em VSVD com balonamento sequencial;
- Retirada das guias e introdutores com compressão e encaminhamento para a UTI.

No período pré-implante de *stent* na VSVD, a mediana do gradiente sistólico máximo na VSVD foi de 63,5 mmHg (variando de 52 a 97 mmHg), enquanto 46 foi a mediana do gradiente sistólico médio na VSVD, que teve variação de 38 a 56 mmHg. A estenose na via de saída do ventrículo direito de predomínio infundibular foi encontrada em quatro dos seis pacientes, prevalecendo sobre à estenose do tipo valvar. Pode-se observar que a mediana do índice de Nakata da artéria pulmonar foi de 96,5 mm (sendo o menor valor de 68,44 e maior de 138 mm), ao passo que a saturação de oxigênio variou de 75% a 90% durante o período pré-IS. Com relação ao tamanho dos ramos pulmonares direito e esquerdo, tronco pulmonar e anel valvar pulmonar, verificaram-se medianas de 3,5 mm (2,1 mm a 4,8 mm), 3,5 mm (2 mm a 5 mm), 6,9 mm (3,5 mm a 7,5 mm) e 4,2 mm (3,5 mm a 6,5 mm), respectivamente. A respeito do peso dos pacientes selecionados, a mediana foi de 4,9 kg, com peso mínimo de 4,0 kg e máximo de 8,3 kg no momento do implante do *stent* . Tais variáveis estão descritas na 3, anexo A.

No período pós-operatório imediato de IS na VSVD, observou-se variação no gradiente sistólico máximo na VSVD de 28 mmHg a 72 mmHg, correspondente a mediana de 50,5 mmHg. Por outro lado, três pacientes apresentaram informações acerca da gravidade de estenose na VSVD em leve, moderada e residual importante. Com relação ao tamanho dos ramos pulmonares direito e esquerdo, verificaram-se medianas de 4,9 mm (2,5 mm a 6,0 mm) e 4,3 mm (2,8 mm a 5,2 mm), respectivamente. Quanto ao índice de Nakata da artéria pulmonar, observou-se que a mediana foi de 108,6 mm (42,44 a 138 mm) e constatou-se mediana de 93% (68% a 98%) na saturação de oxigênio. No que diz respeito ao peso dos pacientes, obteve-se mediana de 5,5 kg, em que o menor peso observado foi de 4,9 kg e o maior, 8,5 kg, conforme descrito na Tabela 1, do material suplementar.

No período pós-operatório tardio da retirada do implante de *stent* e cirurgia corretiva total, o gradiente sistólico máximo na via de saída do ventrículo direito variou entre 17,4 mmHg a 85 mmHg, com mediana de 50,5 mmHg. A mediana do gradiente sistólico médio na via de saída do ventrículo direito foi de 19 mmHg, oscilando entre 10 mmHg a 51 mmHg. O peso dos pacientes atingiu uma mediana de 11 kg, com peso mínimo de 6,5 kg e máximo de 16,5 kg, conforme descrito na Tabela 3 .

**Tabela 3:** – Distribuição das variáveis antropométricas e ecocardiográficas do período pós-operatório tardio de retirada de *stent* na VSVD.

Paciente	Gradiente sistólico	Gradiente sistólico	Peso
	máximo na VSVD	médio na VSVD	(kg)
	(mmHg)	(mmHg)	
A	24	–	16,5
B	41	19	10,1
C	–	–	6,5
D	17,4	10	11
E	85	51	8
F	–	–	–
Mediana	50,5	19	11

*VSVD: via de saída do ventrículo direito.*

Em todos os casos, foi possível implantar o *stent* na VSVD; no entanto, dois casos obtiveram evolução desfavorável. O paciente E apresentou embolização do *stent* 28 horas após o procedimento e necessitou de cirurgia de emergência para substituição da endoprótese, evoluindo para um quadro instabilidade hemodinâmica e óbito. O paciente F foi a óbito 72 horas depois do IS, por causa não relacionada ao procedimento. A mortalidade global foi de 33% (2/6). No que diz respeito a outras complicações relacionadas ao procedimento, o paciente A evoluiu com distúrbio de condução do ramo direito; o paciente C apresentou bradicardia no momento da colocação do *stent,* revertida com atropina, além de revelar estenose ostioinfundibular abaixo da endoprótese, tendo em vista a migração da mesma.

## Discussão

Em 1969, Dotter propôs o implante endovascular de uma prótese que promovesse sustentação luminal, que foi chamada de *stent* . Desde então, é amplamente descrito o uso de endopróteses; porém, há poucos relatos de seu uso em obstruções musculares intracardíacas, como é o caso da anatomia de portadores da TF, compreendida por hipoplasia, atresia pulmonar e/ou estenoses distais dos ramos pulmonares.^7 - 9^

Uma variante paliativa ao IS na VSVD para cardiopatias congênitas é o implante de *stent* ductal, descrito e publicado por Gibbs em 1991 como uma alternativa não cirúrgica para assegurar o fluxo pulmonar em RN e lactentes.^2^ Ele é indicado em situações de fluxo pulmonar dependentes de canal arterial ou quando há restrição de fluxo pulmonar infundibular, mas cujo quadro não está relacionado à valva pulmonar ou à comunicação interventricular, como é habitual na TF4.^7 , 10^ O IS no canal arterial pode exacerbar a estenose infundibular preexistente e gerar distorção do ramo da artéria pulmonar, o que traria efeitos negativos no tratamento definitivo dos pacientes com T4F.^7 , 11^

Sandoval et al.,^11^ em suas análises, definiram que o resultado imediato do implante de *stent* do canal arterial é o sucesso em mais de 80% dos casos, mortalidade precoce entre 0-10% e efetividade no crescimento da árvore vascular pulmonar, principalmente nos casos em que a VSVD está severamente comprometida pela estenose.^11^ Roshental et al.,^12^ em estudo, determinaram que a médio e longo prazo, o implante de *stent* no canal arterial, quando comparado ao BT e à fistula sistêmico-pulmonar (tipo BT modificada), revela uma taxa de reestenose alta em 43% dos pacientes, além de proliferação neointimal no centro *intrastent* e nas bordas aórtica e pulmonares.^11 , 12^ Outra desvantagem reconhecida do implante de *stent* no canal arterial traz referência ao risco de obstrução da luz do canal de até 75% em seis meses após a intervenção paliativa – tempo reduzido quando comparado ao IS na VSVD.^12^

**Figura 1:**
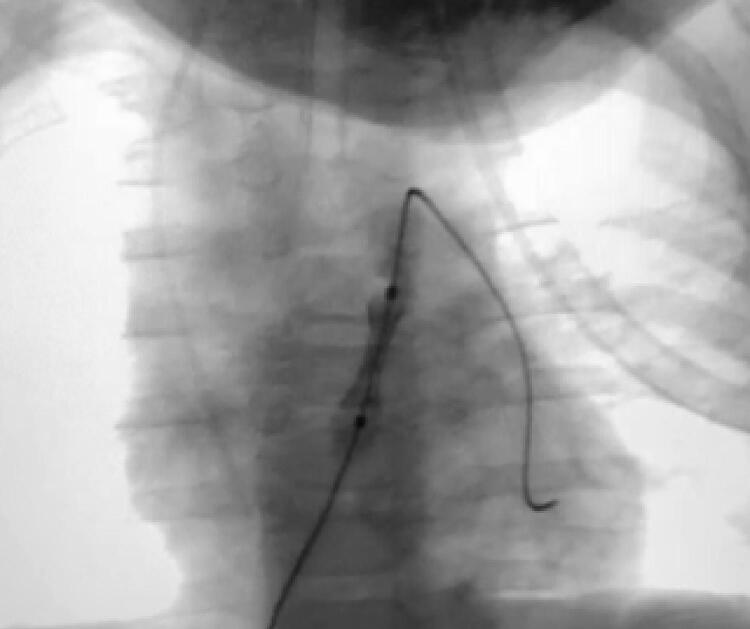
– *Implante de stent na VSVD. Cineangiografia da paciente A: início da dilatação do balonete.*

**Figura 2:**
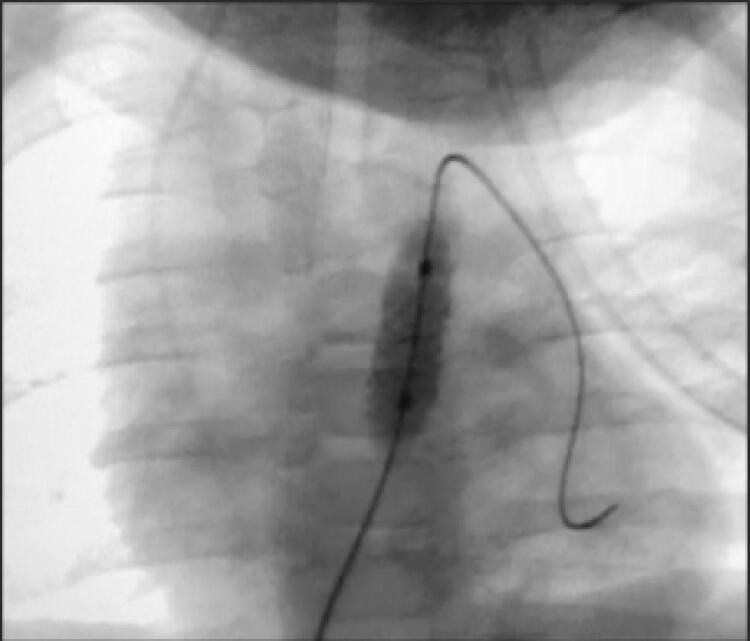
– *Implante de stent na VSVD. Cineangiografia da paciente A: final da dilatação do balonete e posicionamento do stent.*

Apesar de alguns pacientes apresentarem estenose predominantemente valvar, todos apresentavam componente infundibular dinâmico de estenose também importantes, o que faz com que a realização apenas da valvoplastia com balão tenha resultados insatisfatórios.^10 , 13^ Assim, optou-se pelo implante de *stent* na VSVD ( Figuras 1 , 2 e 3 ).

*Fonte: Serviço de Hemodinâmica. Hospital infantil de grande porte em Curitiba (PR).*

*Fonte: Serviço de Hemodinâmica. Hospital infantil de grande porte em Curitiba (PR).*

**Figura 3:**
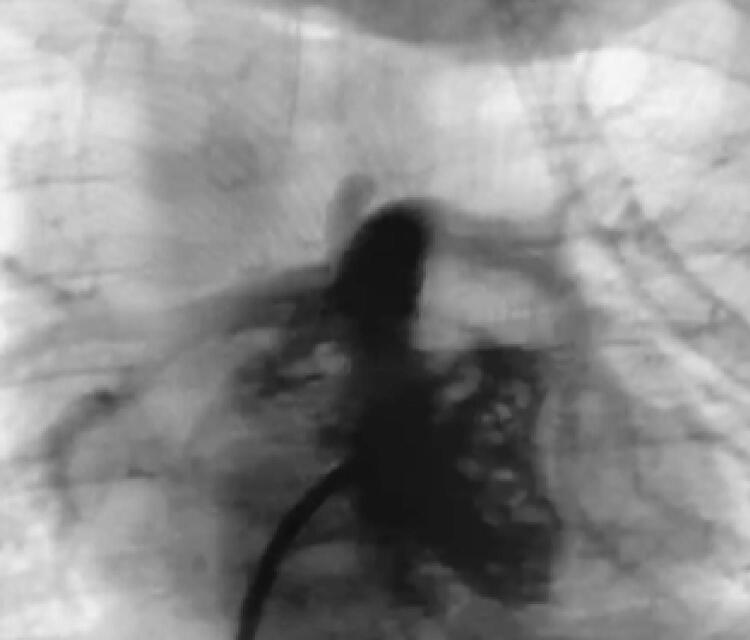
– *Pós-implante de stent na VSVD. Cineangiografia da paciente A: resultado final do procedimento.*

*Fonte: Serviço de Hemodinâmica. Hospital infantil de grande porte em Curitiba (PR).*

No presente estudo, foi possível observar que a mediana de idade dos pacientes foi de 146,5 dias no procedimento de implante de *stent* (IS), enquanto que na retirada associada à cirurgia corretiva foi de 367 dias, com permanência da endoprótese em um período de cerca de 216 dias, bem como as pesquisas disponíveis na literatura indicam idade precoce nos pacientes submetidos a processos de IS e adiamento adequado temporal para efetuar a cirurgia definitiva.^9 , 13 - 15^ Foi possível observar o ganho ponderal adequado dos pacientes para a idade, proporcional ao tempo de permanência do *stent* , o que permitiu o crescimento e o desenvolvimento que promoveu maior sobrevida em procedimento cirúrgico definitivo invasivo. Além de ampliar a sobrevida no pós-operatório, o implante de *stent* na VSVD dispõe de outras vantagens: otimiza o intervalo de tempo necessário para a correção definitiva, restringe a quantidade de cirurgias paliativas mandatórias e restabelece o desenvolvimento neuronal adequado e a qualidade de vida dos pacientes até a cirurgia corretiva definitiva.^6 , 7 , 9^

Há um grande espectro estrutural de variação anatômica da estenose na VSVD na TF quanto à origem da obstrução, como: infundibular, valvar pulmonar, arterial pulmonar e/ou ramificações da artéria pulmonar. Foi observado no presente estudo que os dois primeiros pacientes (A e B) possuiam estenoses de predomínio valvar, enquanto os demais eram portadores de estenoses infundibulares, manifestando à dominância corroborada pela literatura – a exemplo da pesquisa de Costa et al. de 2016, que avaliou 30 pacientes portadores de TF com IS na VSVD e obteve frequência de 43% em casos de estenose com origem infundibular.^9 , 13 - 15^

No ano de 2019, acerca das diferentes intervenções terapêuticas possíveis em pacientes portadores de TF, Sandoval et al.^11^ compararam quatro grupos: um deles realizou IS na VSVD, dois eram formados por pacientes com menos de três meses de idade realizaram a cirurgia corretiva precocemente – dentre eles, um grupo com estenose pulmonar e outro com atresia pulmonar – e o último realizou a cirurgia corretiva em pacientes de três a 11 meses. Os lactentes submetidos ao IS na VSVD expressaram os menores índices de Nakata quando comparados aos outros três grupos, com valores inferiores a 100 mm^2^ /m^2^ .

Tal circunstância esteve presente em nosso estudo: é o caso dos pacientes A e B no período pré-IS na VSVD, que apresentaram, respectivamente, 68,4 mm^2^ /m^2^ e 82 mm^2^ /m^2^ . No período pós-IS, houve incremento relevante nestes índices, o que os elegeu como bons candidatos ao procedimento de correção total da TF por apresentarem índices acima de 100 mm^2^ /m^2^ .^5 , 16^

Quanto às marcas dos *stents* implantados, em nosso estudo foi possível notar predomínio da Dynamic-biotronik sobre a Woven-NIH em três dos quatro procedimentos descritos, os quais apresentaram variação de diâmetro e comprimento conforme estenose na VSVD, salvo registro de especificações técnicas ausentes em dois pacientes.

A mediana do gradiente sistólico máximo na VSVD, no período de pré-implante de *stent,* diminuiu de 69,4 mmHg para 50,5 mmHg após o implante imediato da endoprótese, circunstância descrita na literatura por Peng et al. em 2006 e por Ovaert et al.^17^ em 1999, o que corrobora a presença imediata de queda no gradiente sistólico máximo e de sobrecarga pressórica de VD – objetivo fundamental almejado após a IS na VSVD.^14 , 17^

Os resultados dos ecocardiogramas transtorácicos constataram a taxa de crescimento dos ramos pulmonares direito e esquerdo, pré-implante e pós-implante imediato de *stent* na VSVD. Observou-se também que a mediana dos tamanhos pulmonares direito e esquerdo pré-IS eram de 3,5 mm, ao passo que no pós-IS tais valores subiram para 4,9 mm e 4,3 mm, respectivamente. Destacamos o caso do paciente C, que apresentou desenvolvimento significativo dos ramos pulmonares direito de 3,7 mm para 6 mm, e esquerdo de 3,5mm para 5,2 mm.

Ao comparar os parâmetros de pacientes submetidos ao IS na VSVD com outras opções de procedimentos paliativos ou com a cirurgia corretiva precoce, é possível enquadrar os portadores de *stent* como os detentores dos piores prognósticos: baixo peso ao nascimento, menor índice de Nakata, má anatomia e ramos de artéria pulmonar de pequeno tamanho – características capazes de gerar maior risco de sequelas como a hipoplasia pulmonar. Este fato também foi descrito em 2019 na série de casos de Bigdelian et al.,^5^ que fez uma comparação entre três grupos: oito pacientes que passaram pelo IS na VSVD, sete pacientes que foram submetidos à técnica de BT e 15 pacientes que passaram por por cirurgias corretivas definitivas e precoces. Nesse estudo, foi possível observar que mesmo com menores tamanhos de ramos pulmonares iniciais, os pacientes com *stent* obtiveram desenvolvimento pulmonar comparável ao dos pacientes submetidos à técnica BT e crescimento ponderal similar aos que foram submetidos a outros procedimentos. Isto posto, o crescimento dos ramos pulmonares, bem esclarecido por Bigdelian et al. em 2019, reflete diretamente no aumento da saturação de oxigênio, assim como na melhora da condição hemodinâmica desses pacientes críticos.^5^

Entre as informações disponíveis nos prontuários sobre a saturação de oxigênio capilar, quatro pacientes apresentaram valores compreendidos entre 75% e 90% no período de pré-IS na VSVD. Contudo, no período de pós-IS na VSVD imediato, foi observada a melhora substancial em quatro dos cinco pacientes com informações descritas, que apresentaram valores superiores a 89% – objetivo almejado quando ocorre à indicação do IS.^14^ Um paciente teve complicações no período pós-IS na VSVD imediato, com saturação compreendida em 68%. Nos estudos publicados, não foram descritos grau e duração da cianose, ou sobre a existência de crises de hipóxia nos períodos de pré-IS na VSVD e pós-IS na VSVD imediato.^5 , 13 , 14 , 16^

São raras as complicações descritas na literatura médica. Dentre elas, as mais preocupantes envolvem casos de trombose e mal posicionamento da endoprótese, o que pode acarretar a necessidade de remoção e/ou de substituição cirúrgica do *stent* .^14 , 17^ No presente estudo, dois pacientes foram a óbito, um deles por embolização do *stent* após 28 horas do implante, não havendo êxito na retirada e reimplante. O outro paciente apresentava histórico de transplante hepático e atresia de vias biliares, não resistindo às intercorrências clínicas e veio a falecer 72 horas após o IS na VSVD. No paciente C, não foi possível o adequado posicionamento do *stent* , o que refletiu em estenose ostioinfundibular abaixo da endoprótese. No entanto, não houve repercussões significativas, tendo em vista a ampliação dos ramos pulmonares direito e esquerdo, de 3,7 mm para 6 mm e de 3,5 mm para 5,2 mm, respectivamente; e redução no gradiente sistólico máximo na VSVD, de 69 mmHg no período pré-IS para 40 mmHg no pós-IS de VSVD imediato.

Este estudo apresenta limitações pelo tamanho da amostra e pela presença de registros incompletos das variáveis ecocardiográficas, hemodinâmicas e dos desfechos clínicos nos prontuários relacionados.

## Conclusão

Apesar do pequeno número de pacientes, conclui-se que a intervenção endovascular na VSVD por meio de *stent* é uma medida paliativa eficaz, capaz de retardar a necessidade de intervenção cirúrgica imediata, prolongar a sobrevida dos pacientes com TF e tornar possível a correção cirúrgica definitiva nos lactentes de baixo peso e má anatomia.

## * Material suplementar

Para informação adicional, por favor, clique aqui.
