# Supplementary material for: Intervenção Paliativa Endovascular no Lactente com Tetralogia de Fallot: Uma Série de Casos
Source: Arq Bras Cardiol. 2021 Jul 7;117(4):657–63. [Article in Portuguese] doi: 10.36660/abc.20200148 (PMC8528376; doi:10.36660/abc.20200148)
Supplement: Translation

# Translation: Palliative Endovascular Intervention in Infants with Tetralogy of Fallot: A Case Series

**Authors:** Kerli Dreier Kupas, Isabela Oldoni, Juliano Mendes Souza

**Affiliations:** 1 Brasil Faculdades Pequeno Príncipe, Curitiba, PR - Brasil; 2 Brasil Universidade federal do Paraná (UFPR), Curitiba, PR – Brasil

**Keywords:** Heart Defects, Congenital, Tetralogy of Fallot, Surgical Procedures Operative, Infant, Blalock-Taussig Procedure

## Abstract

**Background:**

Endovascular stent placement in the right ventricular outflow tract (RVOT) has been an alternative to Blalock-Taussig (BT) surgery in the treatment of Tetralogy of Fallot (TOF) in symptomatic infants with low birth weight and complex anatomy.

**Objective:**

To evaluate endovascular stent placement in the RVOT as a primary treatment for infants with TOF who are not candidates for BT surgery, and evaluate medium-term outcomes until the stent is removed during corrective surgery.

**Methods:**

Six infants with TOF were treated with RVOT stenting from October 2015 to April 2018. Hemodynamic parameters were compared between the pre- and post-stenting periods.

**Results:**

At the time of stenting, participants had a median age and weight of 146.5 days and 4.9 kg, respectively. Peak systolic gradient decreased from 63.5 mm Hg to 50.5 mm Hg, while the diameter of the left and right pulmonary arteries increased from 3.5 mm to 4.9 mm and 4.3 mm, respectively. The Nakata index increased from 96.5 mm to 108.3 mm; weight increased from 4.9 kg to 5.5 kg; and oxygen saturation, from 83.5% to 93%. There was one case of stent migration and two deaths, one caused by stent embolization and the other unrelated to study procedures.

**Conclusions:**

RVOT stenting is a promising alternative for the palliative treatment of TOF in infants with low birth weight and complex anatomy.

## Introduction

Tetralogy of Fallot (TOF) is the most common cyanotic congenital heart defect, affecting three in every 10,000 live births worldwide, and occurring more frequently in males.^1 , 2^ It is characterized by four basic anomalies: interventricular communication, obstructed right ventricular outflow tract (RVOT), right ventricular hypertrophy and dextroposition of the aorta.^1 , 2^ The symptomatology varies depending on the degree of stenosis in the RVOT and the extent of intraventricular communication, but typical manifestations include bluish-purple discoloration of the skin, fatigue during feeding, and episodes of hypoxia relieved by squatting.^3 , 4^ TOF can be diagnosed during pregnancy through morphological ultrasound and should be confirmed after birth using fetal or transthoracic echocardiography. In inconclusive cases or during preoperative assessment, procedures such as catheterization, magnetic resonance imaging, or computed tomography can also be helpful.^3 , 5^

Treatment alternatives include corrective surgery, possibly preceded by palliative procedures such as the placement of a Blalock-Taussig (BT) shunt, and RVOT stenting.

BT shunts have been the treatment of choice for FOT since 1945. The procedure involves the surgical placement of a systemic-pulmonary shunt between the subclavian and pulmonary arteries. In neonates and infants younger than 3 months, this procedure is associated with high rates of shunt occlusion, death, and destruction of the pulmonary valve annulus.^3^ An alternative treatment for these low-birth-weight infants with complex anatomical defects and moderate to severe symptoms is RVOT stenting,^6^ a less invasive strategy with lower morbidity and mortality rates that can restore neuronal development and improve quality of life until they undergo definitive corrective surgery.^6 - 8^

In light of these observations, this study aimed to evaluate the RVOT stenting as a primary treatment for children with TOF who are not candidates for BT surgery, and evaluate echocardiographic characteristics in the pre- and postoperative periods until the stent is removed during corrective surgery.

## Method

This study was submitted and approved by a research ethics committee under protocol number CAAE 17443119.3.0000.5580. All procedures were conducted per the guidelines and criteria established in National Health Council (Conselho Nacional de Saúde; CNS) Resolution No. 466, issued on December 12, 2012, which stipulates ethical principles for the preservation of data integrity, privacy, and anonymity.

This was an observational, descriptive longitudinal study involving retrospective quantitative data collected at a large pediatric hospital in Curitiba, in the state of Paraná.

Inclusion criteria were having TOF and undergoing palliative endovascular treatment with RVOT stenting. Exclusion criteria were as follows: stenting due to congenital heart disease other than TOF; having a stent in another anatomical region of the heart; having other cardiac malformations.

The present study evaluated a series of 6 consecutive infants with TOF who underwent RVOT stenting between October 2015 and April 2018.

The following data were collected from medical records for subsequent analysis:

- Sociodemographic data: sex, age in days.
- Anthropometric data: weight in kg.
- Clinical data: percent oxygen saturation.
- Echocardiographic data: mean and peak systolic pressure gradients in the RVOT, measured in mm Hg; type and severity of stenosis in the RVOT; and the size of the left and right pulmonary arteries, the pulmonary trunk, and the pulmonary valve annulus, measured in mm; Nakata index of the pulmonary artery, measured in mm.
- Endovascular prosthesis data: brand and size (diameter in mm x length in mm).
- Surgical data: description of the stenting technique.

These outcome variables were collected at 3 different time points: preoperatively, in the immediate postoperative period of RVOT stenting, and in the late postoperative period of stent removal during corrective surgery.

### Statistical analysis

The data was entered into a Microsoft Excel spreadsheet and analyzed using descriptive methods. Results were expressed using median, minimum, and maximum values, as well as percentages.

## Results

The sample consisted of 6 infants, 3 of whom were female while 3 were male. The median age at the time of RVOT stenting was 146.5 days (range, 68-121). At the time of stent removal, the median age of the sample was 367 days, as shown in Table 1 .

**Table 1:** – Median age of patients at the time of RVOT stenting and stent removal during corrective surgery; length of interval between stent placement and removal

Patient	Age at RVOT stenting (days)	Age at stent removal during corrective surgery (days)	Interval between placement and removal of stent in the RVOT (days)
A	261	1211	950
B	68	1078	1010
C	151	367	216
D	74	184	110
W	142	200	58
F	170	-	-
Median	146.5	367	216

*RVOT: Right ventricular outflow tract.*

The manufacturers and sizes of the stents used in the study are shown in Table 2 . The most commonly used brand was Dynamic-Biotronik. In two cases, the manufacturer of the stents used was not indicated.

**Table 2:** – Manufacturer and size of endovascular prosthesis (stent)

Patient	Stent manufacturer	Stent size (diameter mm x length mm)
A	Dynamic - Biotronik	8 x 12
B	Dynamic - Biotronik	8 x 15
C	Woven - NIH	8 x 15
D	Dynamic - Biotronik	7 x 15
E	-	-
F	-	-

*RVOT: Right ventricular outflow tract.*

The stenting technique used in the present study is described below:

- Patient under general anesthesia;
- Placement of 6F and F5 sheaths in the right femoral vein and artery, respectively;
- Judkins catheter advanced over a 0.035” hydrophilic guidewire and manometry performed;
- Collection of serial blood samples for gasometry, oximetry, and measurements of flow and resistance;
- Cineangiography via pigtail catheters;
- Exchange of the 0.035” for a 0.014” guidewire;
- Placement of the stent in the RVOT followed by balloon inflation;
- Removal of guidewires and sheaths, application of compression, and referral to the ICU.

Prior to stenting, the median peak systolic gradient across the RVOT was 63.5 (range, 52-97) mm Hg, while the median value for the mean systolic gradient at the RVOT was 46 (range, 38-56) mm Hg. Infundibular stenosis of the right ventricle was present in 4 out of 6 patients, while valve stenosis was less frequently observed. The median Nakata index for the pulmonary artery was 96.5 (range, 68.44-138) mm, while the oxygen saturation ranged from 75 to 90% during the pre-stenting period. The median size of the left and right pulmonary branches, pulmonary trunk, and pulmonary valve annulus were 3.5 (range, 2.1-4.8) mm, 3.5 (range, 2-5) mm, 6.9 (range, 3.5-7.5) mm, and 4.2 (3.5 - 6.5) mm, respectively. The median weight of patients at the time of stenting was 4.9 (range, 4.0-8.2) kg. These variables are described in Table 3 , appendix A.

**Table 3:** – Distribution of anthropometric and echocardiographic variables in the late postoperative period after stent removal from the RVOT

Patient	Systolic Gradient	Systolic Gradient	Weight
	Peak across RVOT	Mean across RVOT	(kg)
	(mm Hg)	(mm Hg)	
A	24	-	16.5
B	41	19	10.1
C	-	-	6.5
D	17.4	10	11
W	85	51	8
F	-	-	-
Median	50.5	19	11

*RVOT: Right ventricular outflow tract.*

In the immediate post-operative period after stenting, the peak systolic gradient across the RVOT in the sample ranged from 28 to 72 mm Hg, with a median value of 50.5 mm Hg. However, the records of 3 patients revealed the presence of mild, moderate, and significant residual stenosis in the RVOT. The median sizes of the left and right pulmonary branches were 4.9 (range, 2.5-6.0) mm and 4.3 (2.8-5.2) mm, respectively. The median Nakata index for the pulmonary artery was 108.6 (range, 42.44-138) mm, while median oxygen saturation was 93% (range, 68-98%). Lastly, at this time, the median weight in the sample was 5.5 (range, 4.9-8.5) kg, as described in Table 1 , supplementary material.

In the late postoperative period after stent removal and corrective surgery, the peak systolic gradient across the RVOT ranged from 17.4 to 85 mm Hg, with a median value of 50.5 mm Hg. The mean systolic pressure gradient had a median value of 19 (range, 10-51) mm Hg. Patients weighed a median of 11 (range, 6.5-16.5) kg, as described in Table 3 .

In all cases, the stent was successfully inserted in the RVOT, although 2 cases had an unfavorable clinical course. Patient E experienced stent embolization 28 h after implantation, requiring emergency surgery to reposition the stent. This was eventually followed by hemodynamic instability and death. Patient F died 82 h after stenting for causes unrelated to the procedure. The overall mortality rate in the present study was 33% (2/6). With regard to other complications observed after the procedure, Patient A displayed a right bundle branch block, while Patient C had bradycardia during stent placement, which was reversed with atropine, as well as infundibular stenosis below the graft due to stent migration.

## Discussion

In 1969, Dotter developed a technique involving the endovascular implantation of a prosthetic device to support the structure of the venous lumen. The device was referred to as a stent. Since then, studies have evaluated the use of vascular prostheses in a variety of cases, although few focused on patients with intracardiac flow obstruction as seen in TOF, whose manifestations include pulmonary atresia and/or hypoplasia, and distal pulmonary branch stenosis.^7 - 9^

A palliative alternative to RVOT stenting for the treatment of congenital heart disorders is the placement of a duct stent, described in a publication by Gibbs in 1991 as a non-surgical alternative to ensure adequate pulmonary flow in neonates and infants.^2^ This technique is indicated for cases of ductus arteriosus-dependent pulmonary flow or infundibular pulmonary stenosis not associated with the pulmonary valve or interventricular communication, as in the case of TOF.^7 , 10^

Stenting the ductus arteriosus can exacerbate infundibular stenosis and lead to deformation of the pulmonary artery so that its use in the treatment of patients with TOF would have several negative consequences.^7 , 11^

In a study of the immediate results of arterial stent placement, Sandoval et al.^11^ found the procedure to be successful in over 80% of cases, with early mortality rates of 0-10% and effectiveness in promoting the growth of the pulmonary vascular tree, especially in cases of severe stenosis of the RVOT.^11^ Rosenthal et al.^12^ determined that in the medium- and long-term, arterial stents are associated with a higher likelihood of restenosis relative to BT and systemic-pulmonary fistula (modified BT shunt); arterial stents are associated with a restenosis rate of 43%, in addition to neointimal proliferation in the intrastent segment and the aortic and pulmonary borders.^11 , 12^ Another known limitation of arterial stents is the risk of luminal obstruction, which can reach 75% in the 6 months after the palliative intervention - a much shorter time than observed in RVOT stenting.^12^

Though some patients had predominantly valvular stenosis, all showed a significant degree of dynamic infundibular obstruction, so that balloon valvuloplasty alone would have unsatisfactory results.^10 , 13^ We therefore opted for RVOT stenting ( Figures 1 , 2 , and 3 ).

**Figure 1:** – *Stent implantation in the right ventricular outflow tract. Cineangiography of Patient A: start of balloon expansion.*

*Source: Hemodynamics service. Large pediatric hospital (Curitiba - PR).*

**Figure 2:** – *During stent implantation in the right ventricular outflow tract. Cineangiography of patient A: end of balloon expansion and stent positioning.*

*Source: Hemodynamics service. Large pediatric hospital (Curitiba - PR).*

**Figure 3:** – *After stent implantation in the right ventricular outflow tract. Cineangiography of Patient A: final result of the procedure.*

*Source: Hemodynamics service. Large pediatric hospital (Curitiba - PR).*

In the present study, the median age of patients at the time of stent placement was 146.5 days, while at the time of removal and corrective surgery, patients had a median age of 367 days. The time spent with the stent in place was approximately 216 days. This is in line with previous research, which highlights the early age of patients submitted to stenting, and the need to wait for a sufficient time before performing definitive surgery.^9 , 13 - 15^ It was also possible to confirm that patients had adequate weight gain for their age, proportional to the time with the stent, allowing for sufficient growth and development to improve survival after the invasive surgical procedure. In addition to improving postoperative survival, RVOT stenting has other advantages such as optimizing the time interval for definitive surgery; reducing the number of palliative surgeries required; and restoring neuronal development and quality of life until corrective surgery.^6 , 7 , 9^

The anatomy of RVOT stenosis can vary significantly between patients concerning the origin of the obstruction, which can be in the infundibulum, the pulmonary artery, and/or the pulmonary artery branches. In the present study, the first two patients (A and B) had predominantly valvar stenosis, while the other patients had infundibular stenosis. These findings reflect the results of previous studies, such as that of Costa et al., 2016, who found that 43% of a sample of 30 patients with TOF who underwent RVOT stenting had infundibular stenosis.^9 , 13 - 15^

Concerning the different treatment possibilities for patients with TOF, Sandoval et al.,^11^ compared four groups. One underwent RVOT stenting; two groups with patients younger than 3 months—one group with pulmonary stenosis and the other with pulmonary atresia—underwent early corrective surgery; and the last underwent corrective surgery at 3 to 11 months of age. Infants submitted to RVOT stenting had lower Nakata indices than those in all other groups, with values below 100 mm^2^ /m^2^ . This was also observed in the present study, with Patients A and B showing indices of 68.4 mm^2^ /m^2^ and 82 mm^2^ /m^2^ in the pre-stenting period, respectively. In the post-stenting period, these values increased significantly and exceeded 100 mm^2^ /m^2^ , confirming that participants were good candidates for total correction of TF.^5 , 16^

Regarding the manufacture of the stents used in the present study, 3 of the 4 patients received devices produced by Dynamic-Biotronik, while one patient received an endoprosthesis made by Woven-NIH. The devices varied in length and diameter depending on the degree of stenosis in the RVOT. This information was unavailable for 2 patients.

The median peak systolic gradient across the VSOT decreased from 69.4 mm Hg in the pre-implant period to 50.5 mm Hg in the immediate post-implant period, following a similar pattern to that described by Peng et al., 2006, and Ovaert et al.,^17^ 1999, who observed a decrease in the peak systolic gradient and right ventricular pressure overload immediately after surgery, confirming that the procedure had fulfilled its main purpose.^14 , 17^

Transthoracic echocardiography also showed the growth rate of the left and right pulmonary branches before and immediately after the placement of the stent in the RVOT. The median sizes of the right and left pulmonary arteries prior to stenting were 3.5 mm, but increased to 4.9 mm and 4.3 mm, respectively, after the procedure. These changes were especially evident in Patient C, whose right pulmonary artery increased in size from 3.7 mm to 6 mm, while the left went from 3.5 mm to 5.2 mm. Patients submitted to RVOT stenting rather than other palliative procedures or early corrective surgery usually exhibit risk factors for poor prognosis, such as low birth weight; lower Nakata index; anatomical malformations small pulmonary artery branches, which have an increased risk of complications such as pulmonary hypoplasia. This was also observed in a case series by Bigdelian et al.,^5^ 2019, who compared 3 groups: 8 patients who underwent RVOT stenting, 7 who underwent BT surgery, and 15 who underwent early corrective surgery. Even though patients with stent placement initially had smaller pulmonary artery branches than the other participant groups, their lung development was similar to that of patients who underwent BT surgery, while their weight gain did not differ from that of the other participants. As described by Bigdelian et al., 2019, the increased size of pulmonary artery branches results in improvements to the oxygen saturation and hemodynamic status of these critical patients.^5^

The information available on patient records showed that 4 participants had oxygen saturation values ranging from 75 and 90% prior to stent placement. However, immediately after stenting, there was a substantial improvement in 4 of the 5 patients for whom this information was available. The 4 participants showed values over 89%, indicating that stenting was successful;^14^ one patient, however, experienced complications after stenting and had a saturation of 68%. No information was available on the degree and duration of cyanosis or the presence of hypoxia before and immediately after stenting in previously published studies.^5 , 13 , 14 , 16^

Complications are rarely described in the literature, but the most concerning include thrombosis and malpositioning of the endoprosthesis, which may require surgical removal and/or replacement of the *stent.* ^14 , 17^ In this study, 2 patients ultimately died. One death was associated with the occurrence of stent embolization 28 hours after stent placement, which was followed by unsuccessful attempts at stent removal and reimplantation. The other patient had undergone a liver transplant and had biliary atresia. The patient experienced complications and died 72 hours after stenting. In the case of Patient C, the stent could not be adequately positioned, resulting in infundibular stenosis below the placement site. However, this had no significant clinical consequences, with the patient showing an increase in the size of the right and left pulmonary artery branches - from 3.7 to 6 mm and 3.5 to 5.2 mm, respectively - and a reduction in peak systolic gradient across the RVOT, which fell from 69 mm Hg before stenting to 40 mm Hg in the immediate period after stent placement.

Limitations of this study include the sample size and a lack of data on echocardiographic variables, hemodynamic parameters, and clinical outcomes due to incomplete medical records.

## Conclusion

Despite the small sample size, the present study demonstrated that an endovascular intervention consisting of the placement of a stent in the RVOT was effective at delaying the need for immediate surgical intervention, extending the survival of patients with TOF, and allowing for the definitive surgical correction of congenital disorders in low-birth-weight neonates with anatomical defects.

## * Supplemental Materials

For additional information, please click here.
